# Supplementary material for: Estimating Overall and Cause-Specific Excess Mortality during the COVID-19 Pandemic: Methodological Approaches Compared
Source: Int J Environ Res Public Health. 2023 May 24;20(11):5941. doi: 10.3390/ijerph20115941 (PMC10252246; doi:10.3390/ijerph20115941)
Supplement: Supplementary file 1 [file ijerph-20-05941-s001.zip › ijerph-2186140-supplementary.pdf]

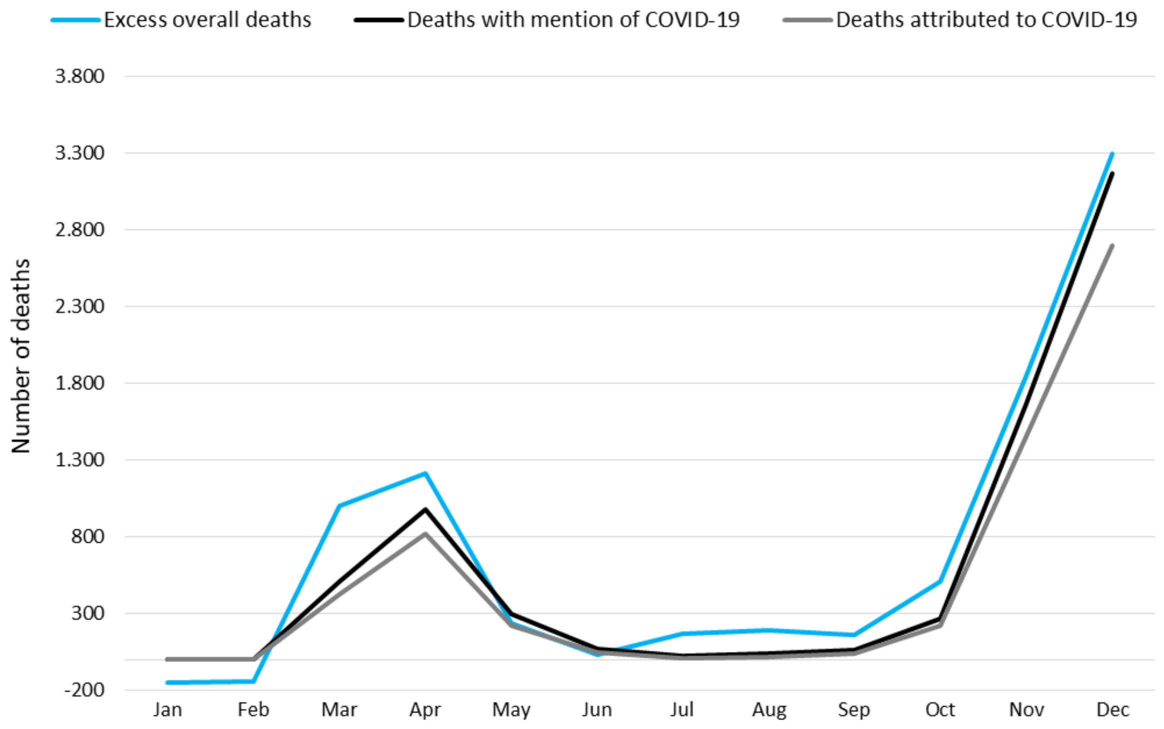

Supplementary Figure S1. All-cause excess deaths (approach based on *number of deaths*), deaths with mention of COVID-19 and deaths attributed to COVID-19 for all causes by month of death during 2020 (Veneto Region, Italy).
